# The smart meter and a smarter consumer: quantifying the benefits of smart meter implementation in the United States

**DOI:** 10.1186/1752-153X-6-S1-S5

**Published:** 2012-04-23

**Authors:** Brendan Cook, Jerrome Gazzano, Zeynep Gunay, Lucas Hiller, Sakshi Mahajan, Aynur Taskan, Samra Vilogorac

**Affiliations:** 1 University of Chicago, Chicago, IL, USA

## Abstract

The electric grid in the United States has been suffering from underinvestment for years, and now faces pressing challenges from rising demand and deteriorating infrastructure. High congestion levels in transmission lines are greatly reducing the efficiency of electricity generation and distribution. In this paper, we assess the faults of the current electric grid and quantify the costs of maintaining the current system into the future. While the proposed “smart grid” contains many proposals to upgrade the ailing infrastructure of the electric grid, we argue that smart meter installation in each U.S. household will offer a significant reduction in peak demand on the current system. A smart meter is a device which monitors a household’s electricity consumption in real-time, and has the ability to display real-time pricing in each household. We conclude that these devices will provide short-term and long-term benefits to utilities and consumers. The smart meter will enable utilities to closely monitor electricity consumption in real-time, while also allowing households to adjust electricity consumption in response to real-time price adjustments.

## Background

The current electrical grid system in the United States is technologically outdated and does not efficiently meet today’s demand for electricity. The grid was built in the 1960s and was not designed to support current levels of electricity consumption. It is important to note the main problems with the electric grid are not primarily due to a shortage of electricity production capacity. While there is reason for additional generation capacity and changing the mixture of energy sources, the problems arise primarily from failures to efficiently deliver generated electricity to end customers.

Electricity itself is a secondary source of energy and is produced by primary sources of energy such as coal, natural gas and wind. Currently, almost half of the electricity generation in the United States comes from coal-burning power plants, followed by natural gas and nuclear power. Electricity generation from renewable sources accounts for approximately 10% of total electricity generation in the United States [1]. In total, there approximately 5,700 power facilities operating the United States, with a nameplate generation capacity of at least one Megawatt [2].

Coal-burning power plants have been more popular due to lower production costs and the abundance of coal, which leads to lower electricity prices. However, these plants have a high capital cost and long construction time. Electricity production is responsible for approximately 35 percent of all greenhouse gas emissions in the United States, amounting to 2,291.8 metric tons of carbon dioxide equivalent in 2009 [3].

Natural gas power plants are promising energy source in the sense that natural gas power plants have a lower cost of capital and shorter construction time. Current capacity additions indicate that there is an increasing trend in natural gas power plant investments. In the absence of sufficient domestic resources, however, increased electricity generation from natural gas could make the United States more vulnerable to shocks to world natural gas markets, international prices and imports [4].

Renewable energy sources are another option that has become more popular recently due to the softer impact on the environment and, in some cases, low production costs. However, physical and regulatory constraints have prevented renewable energy from becoming a primary source of electricity generation. The biggest constraint is the lack of the necessary transmission network that would allow this new electricity supply to be carried from remote areas to high demand centers.

There are three major power grids operating in the 48 contiguous states. These grids generally operate independently of each other, although there are limited links between them. Major areas in Canada are totally interconnected with our Western and Eastern power grids, while parts of Mexico have limited connection to the Texas and the Western power grids. Besides these three major interconnected systems, there are a large number of other operational institutions such as utilities, regulatory agencies or state-run facilities. The fragmented nature of the grid prevents efficient energy distribution and makes it especially difficult to introduce new legislation and infrastructure improvement projects.

### Inefficiencies of the current grid

Outdated technology and insufficient investment hinders the grid’s ability to operate in an efficient, reliable, and environmentally sound manner. Major problems of the current grid are as follows:

#### Congestion rent

Congestion occurs when the quantity of electricity demanded at a particular time is more than what the transmission lines can deliver. It can also occur when the government enforces operational restrictions on the amount of electricity that can be transferred. Congestion rent is at its highest when transmission lines are already heavily loaded, as it becomes more costly to push through an additional unit of electricity. Thus, the more loaded the lines are, the more expensive it is to deliver more electricity.

#### Higher prices

In addition to congestion rent, there is another price effect that results from transmission congestion. Congested lines impede efficient electricity flow in the sense that they prevent electricity from being delivered from cheaper generation facilities to end customers. Thus, when the lines are heavily loaded, electricity might have to be delivered from higher-cost electricity suppliers, which results in higher prices for customers.

#### Power outages

Congested transmission paths also result in power disturbances and blackouts, which can occur as a short or long term loss of electricity to a particular geographic area. The severity of a blackout depends on many factors such as the duration, location and time of day. In addition to congestion, power disturbances also occur due to transmission lines that are susceptible to severe weather conditions, and animal and human interference.

#### Line losses

Transmission and distribution losses in the United States accounted for about 5 percent of all electricity produced in 1970, and have grown to approximately 9.5 percent in 2001. Currently, electricity lost solely in transmission lines is around 10 percent [5]. In this process, generated electricity leaves the power plants but fails to reach end customers. Line losses occur most often in power lines with lower voltages and are mostly due to heavy utilization and congestion.

#### Wasted electricity

Electricity demand is not smooth across time, that is, there are peak and non-peak demand times during the day. However, electricity always has to be available to customers, which requires power plant operators to generate a minimum amount of electricity, ensuring that there is enough excess capacity available at all times. When demand is lower than this amount, which is the vast majority of the time, the unused electricity is simply wasted.

### Estimated costs of the current grid

Below we estimate the size of the annual burden to the economy due to the inefficiencies of the current grid:

#### Congestion rent

Congestion rent is calculated by multiplying the marginal production cost of pushing one more Megawatt hour through a transmission constraint, times the number of Megawatt hours that flow through the constraint, and summing the products for the hours during a year when the constraint is limiting [6]. The National Electric Congestion Study estimates that congestion rent resulted in $8.36 billion in losses for 2009.

#### Line loss

We have stated that 10 percent of electricity generated is lost while it is sent through transmission lines. In 2007, 4,156,745 million kWh of electricity were produced in the US [7]. Thus, the 10 percent loss is equal to 415,674 million kWh. In order to find the dollar value of this quantity, we can use the average cost of producing one kWh of electricity, which is $0.06068. This suggests total annual losses of approximately $25.2 billion.

In order to do the same calculations for year 2009, we need the total electricity produced and the average cost. Projected energy production in 2009 is 4,068,320 million kWh. However the cost of production is unknown at this point, as data is made available every two years. If base our calculations on 2007 nominal prices, we estimate that $24.7 billion is lost due to transmission line losses.

**Figure 1:**
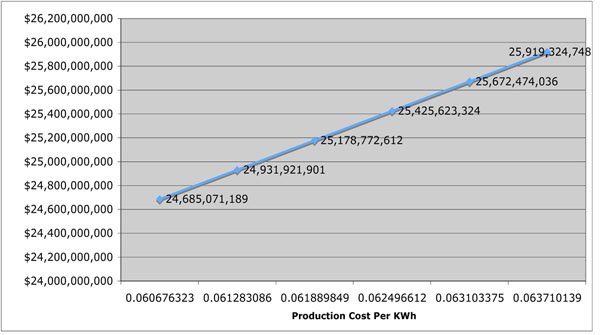
Total U.S. electricity production cost

Energy production has decreased due to recession in 2008 and 2009 however investment in different energy sources continued during previous years, while prices of the primary energy sources have increased, yielding a higher production cost. Thus if we assume that the price of electricity production has increased 1-5% over two years we can see that dollar value of losing 10% of generated electricity increases the economic burden in a major way. A five percent increase in the electricity production cost would increase the cost of loss by more than one billion dollars. Detailed changes can be seen in Figure 1.

#### Cost of unused electricity

We have already calculated electricity wasted in transmission lines. Unused electricity, on the other hand, is the amount of electricity that is generated by not actually consumed by end customers. Figure 1 indicates that 68.5 percent of electricity is wasted in total. Of this, we know that 10 percent is lost solely in transmission, which has a monetary value of $25.2 billion. Based on these statistics, we estimate that total electricity loss has a monetary value of $174.8 billion, of which $149.6 billion is due to unused electricity.

#### Costs of outages

The cost of outages depends on many things such as the number of blackouts in a year, the duration of each blackout, locations, and time of day. There have been attempts to quantify the cost of blackouts yet there seems to be no single formula. The following equation can be used to evaluate the cost of blackouts based on the variables mentioned above [8]:

where *α* is the initial interruption, *β* is the duration adjusted size, *γ* is the geographic scale, *η* is the duration. We can take both *α* and *γ* as positive multipliers since blackout costs increase with both the initial size (MW) and the duration adjusted size (MWh). However, we also expect costs to increase super-linearly with the quadratic terms *β* and *η* due to compounding social costs that come from the scale and duration of a blackout. Estimating the cost of blackouts is beyond the scope of this paper, and we will assume that the annual cost of power disturbances to the economy is $150 billion as estimated by the Galvin Electricity Initiative [9].

#### Carbon emissions

One of the most important social costs of the current system is the emission of greenhouse gases. Expected electricity consumption for 2009 is 4.8 quadrillion btu, which translates it into CO_2_ emissions of 4901.0826 million metric tons. While there are many estimates of social cost of carbon emissions, we assume a conservative price estimate of $25 per metric tons. This means that $1.223 trillion in CO_2_ costs will be emitted in 2009. Due to the growth in electricity consumption this number is expected to grow over the next decade. The total estimated losses are presented together in Table 1.

**Table 1:** Total electric grid losses in 2009

Source of loss	Annual cost
Congestion rent	$8,360,000,000
Line losses	$24,686,565,760
Unused electricity	$149,622,024,900
Power interruptions	$150,000,000,000
CO2 emissions	$1,223,000,000,000

Total cost in 2009	$1,555,668,590,660

### Reducing inefficiency with the current grid

As previous analysis shows, the inefficiency of the current system is a big cost to the economy. There are ways to reduce this inefficiency by improving transmission without radically switching to a new system. The two main ways to do so are current minimization and resistance minimization.

#### Current minimization

The first cause of inefficiency in electricity transmission is the Joule effect. This physical phenomenon corresponds to the heat produced by electric current. For a certain resistance *R* and for a current *I*, the power dissipated by the Joule effect can be calculated as follows:

In order to reduce this loss, we can reduce *I*. We know from Ohm's law that:

The power of the electric signal is:

Therefore, in order to minimize the current *I* without changing the power of the electric signal, the voltage *V* is maximized. That is the reason why high voltage transmission lines are more efficient in transporting electricity. Currently, the voltage typically used is in transmission varies between 50 kV and 1100 kV.

#### Resistance minimization

A transmission line has its own resistance R. It is important to try to reduce it as much as possible since it is a major parameter in the loss by the Joule effect.

where *ρ* is its resistivity, *l* the length of the line and *s* the section.

First, we can reduce the resistance by using more efficient materials. Currently, copper is less and less used, whereas aluminium and steel (on the same line) are considered as the best materials. They have greater resistance than copper, but they are cheaper and lighter, which is important for the construction of transmission lines. The measures of resistance for copper and aluminium-steel combination respectively are as follows: *ρ_Copper_* = 1,72 × 10^-8^ Ω.m ; *ρ_Alumunium_*_+_*_Steel_* = 3 × 10^-8^ Ω.m. We can imagine that this parameter will be improved in the future. With the exploration of new materials and combinations of different materials, the Joule effect with transmission lines could be reduced. However currently relying on more efficient materials is not the best solution since it is more of a long-term and expensive solution while electricity demand is growing rapidly.

Second, we can adjust line diameter and length. In the expression of the resistance of the power line, we cannot change the length, but we can change the section of the line. On first glance, it seems to be a good idea to choose the biggest section possible, since it would reduce the resistance. However, lines with very large diameters pose obvious physical constraints. The *skin effect* becomes a problem when the frequency is too high and when the power line is too large. The electric current flows at an average distance e from the center of the line according to the following equation:

Thus, when the frequency rises, the resistance increases, which is responsible for more energy loss. Clearly, while adjusting the size and length of transmission lines increases efficiency in theory, these methods are perhaps better thought of as long term improvements to the current grid, due to the high cost and time consuming nature of these changes. Thus, it does not seem to be a reasonable approach in solving the immediate problems of the current grid.

We have discussed methods to improve efficiency in the current grid system by focusing on the transmission system. From the two methods mentioned, current minimization and resistance minimization, we will focus on the first, which entails upgrading transmission lines. Transmission lines older than 35 years typically need replacement due to their low efficiency and high risk of causing blackouts. Currently there are 365,058 miles of transmission lines in the United States [10]. We will define: *β* as the percentage of lines that are 35 years old or older, and *α* as the percentage of the (365,058)*β* that needs to be upgraded to a higher voltage level. Thus, (1 – *α*) would be the percentage of lines to be replaced with the same voltage. In addition, in order to minimize congestion rent and the number of congested transmission lines, some lines that have not necessarily completed their life spans should be upgraded to higher voltages as well. This number can be calculated as (1 – *β*)(365,058)*θ*, where *θ* is the percentage of younger lines that needs upgrading due high demand of electricity. Then we can say that the number of lines that needs upgrading or replacing is:

The average cost of upgrading lines to a higher voltage is *P*_1_ = $5.7 million [11]. The average cost of replacing lines with the same voltages is *P*_2_ = $1.28 million [12]. Then the cost to the economy excluding transaction costs due to regulations would be:

Due to the lack of data on the miles of transmission lines that need to be upgraded, we are unable to present a dollar value for upgrading the transmission system to a more efficient one. However we do know that the costs of upgrading even one mile of a transmission line are very high, and there are many lines waiting to be upgraded. This suggests that the overall cost of renewing the transmission system will be very expensive, and it will not solve the immediate problems. We therefore turn to consider electric grid improvements, which can solve the immediate problems while also providing benefits in the long term.

## Methods

The majority of the “smart grid” proposals in the United States focus on two major improvements to the current electric system. The first is the employment of automated metering infrastructure, a new metering technology that allows the grid to communicate with the customer through a device installed in each household. The second is the large-scale improvement of transmission and distribution infrastructure of the electric grid. This includes physical upgrades to the distribution system to improve the reliability, including advanced monitoring devices deployed throughout the grid. The latter improvements would enable utilities to more efficiently identify and solve grid problems. By enabling the household to monitor its electricity consumption, the “smart grid” will bring balance and efficiency to the way in which energy is produced and distributed.

Smart metering provides two-way communication between the consumers and the utility so as to empower the consumer with the information necessary to effectively manage their electricity consumption. It promises significant benefits as it leads to more efficient generation of electricity, improved responsiveness to infrastructure glitches and a reduced load on the grid during peak hours. The smart meters will be installed in each household and will measure the customer’s energy usage in real time as well as inform them of electricity prices in real time. This provides customers with the necessary information required to alter their energy consumption to achieve more efficient and cost-effective energy usage habits.

A primary goal of smart meter implementation is to better know the demand of every consumer, in order to adapt the supply of electricity. The introduction of various informatics devices has made this possible. The amount of electricity a house uses is measured using electricity meters installed in the consumers premise. Information about the electricity consumption from the house is sent to the electricity provider, where it can be analyzed. In order to interact at any time with the electricity provider, the smart meter needs real-time sensors and connection to reliable communication network.

There are many kinds of smart metering solutions [13]. These include Advanced Metering Infrastructure (AMI), Home area networks (HAN), Demand-Response Programs and upgrades to utility information technology architecture and applications that will support “plug-and-play” technology in the future. Advanced Metering Infrastructures (AMI) is the network that creates a two-way communication between the consumers and the utility providers. It is comprised of a “smart meter” at the customer’s premise, a communications network between the smart meter and the utility, and a “meter data management application” (MDMA) at the utility. The consumers are informed about their energy use so that they can use their electricity efficiently as the digital “smart meters” monitor the amount and time of electricity consumption. The communications network sends data to consumers on real pricing and control signals as well as collects information about the smart appliances and devices at consumer’s homes. Real-time pricing would allow utilities to increase prices during peak hours; hence, consumers pay different prices for different periods of the day, and can adjust their consumption accordingly. The MDMA is computer hardware and software that processes the hourly energy usage data.

The biggest challenge to this system is the installation of a good communication network so that there is a smooth transfer of information between the consumer and utility. “Power line communications” is a concept that has been introduced for this purpose. These lines are used to connect to the Internet directly through an electric outlet. This is possible as an electric transmission line can be used not only to carry electricity, but also to carry data or pieces of information. The signal corresponding to this information has a higher frequency than the electrical signal and uses less energy. Because of some attenuation and modifications, it is necessary to repeat the signal many times. This model can be efficiently applied to the communication network between the smart meter and the utility providers. We can observe how the technologies used in the framework of the smart grid are not really new. They are improvements of existing technologies or applications of some techniques for another purpose.

In addition to this technology, there are other solutions that are going to be adopted to enhance consumer involvement in the future electric grid system. Some of these solutions include upgrades to utility information technology architecture and applications that will support “plug-and-play” technology in the future, Home Area Network technology and the Demand –Response programs. “Plug –and –play” technology is the ability to add a new component to a system and have it work automatically without making any manual configurations or doing any technical analysis. Home Area Network is a type of technology that allows consumers to remotely control electronic devices in their houses. The Home Area Network will enable consumers to use their discretion to conserve energy by being able to automatically turn their smart appliances on or off. By reducing peak electricity use during critical periods, the customers themselves can help ensure reliable and affordable electricity at homes and businesses.

The transmission and distribution of electricity depend on a number of individual operators that are responsible for the efficient management of the smart grid. To achieve smooth distribution and transmission there has to be greater interaction among human operators, computer systems, communications networks and data-gathering sensors present at different substations. The goal of the advanced distribution and transmission operations is to improve reliability and enable “self-healing” of the current electric grid. The “self healing” smart grid has three goals [14]. The first goal is to monitor electricity distribution in real-time. Sensors are used to control, measure and manage electrical parameters such as voltage and current to determine the energy used. It enables utilities to monitor, identify and quickly correct problems so as to increase the reliability of power. The second goal is anticipation, which involves looking into the future functionality of the smart grid. The system focuses on identifying any problems that grid is facing and in turn looks for corrective actions and solutions so that potential problems do not cause larger disturbances. The third objective is isolation. The system has the ability to split a potentially larger failure into isolated “islands,” each of which can be solved independently and efficiently. In this way, small outages may take place, but it will prevent major blackouts.

While there are multiple programs within the category of the “smart grid,” we have identified the installation of the smart meter as having many potential benefits with low-cost installation that can be implemented quickly and efficiently. The goal of most smart grid programs is to enable the consumer to more efficiently manage consumption and to enable to utility to efficiently manage production. Because the completion of transmission infrastructure upgrades is both costly and a long-term project, we find that smart meter installation is the best first-step toward a comprehensive improvement of the electric grid in the United States.

## Results

### Elasticity of demand for electricity

The price elasticity of demand for electricity is an essential concept to consider when studying the economics behind electricity demand and the practicality behind the smart grid. For the purpose of this paper, we will focus on electricity production, supply, and consumption at the residential level. In our analysis for price elasticity of demand, we look to analyze how the average US household will react to changes in electricity prices. The purpose of identifying the price elasticity of demand is to determine whether real time pricing is likely to be effective, and if so, the extent to which it will alter electricity consumption at the household level.

The intuition is that the average residential household will adjust their desired quantity of demand of electricity in response to changes in price of electricity. If the price of electricity rises, then customers will reduce the quantity of electricity they consume. Similarly, if the price of electricity decreases, we expect that customers would increase the quantity of electricity they consume. We call the price elasticity of demand the value that relates the responsiveness of customers to changes in the price of electricity.

We can think about the consumer demand for electricity mathematically: we can let x(p,y) be the common electricity demand function of the consumer who faces a constant price p and who receives an income y. Therefore, for this consumer, the optimal consumption of electricity for a consumer who faces nonlinear increasing prices will be defined by x*, and x*= x(p*, y*, z; *β*) where p* is the consumer’s equilibrium marginal willingness-to-pay, y* is the needed income level to produce electricity consumption of x* at the price p*, z is represents observed electricity consumer characteristics, and *β* is a set of parameters to be estimated; however, it makes most sense to view beta as a consumers indifference—a measure between 0 and 1— to consume electricity. For example, the higher the beta, the more indifferent the consumer is about using electricity. This will be helpful for later analysis because when we start to see price elasticity of demand for electricity at an appliance level, beta will change drastically. We would expect that the beta for using a fridge, for example, would be lower than the beta for using a television set or a video game, as the consumer is more indifferent about the luxury of television than keeping their food cold.

Before we analyze the price elasticity of demand for electricity we expect for electricity, it is important to understand the math behind the value. As we stated, the value for the price elasticity of demand measures the rate of response of quantity demanded due to a change in price. The formula for the price elasticity of demand (*ε_d_*) is as follows is as follows:

When the absolute value of the price elasticity of demand is greater than one, we see that the percentage change in the quantity of electricity demanded is greater than that the percentage change in the price, or, in other words, the demand is highly sensitive to price changes. However, we expect that the price elasticity of demand for electricity will be negative, which means that the percent change in the quantity of electricity demanded and the percent change in price change in opposite directions.

When we analyze the price elasticity of demand for electricity, it is important to note that we are doing so on the “short-term” time frame. To explain why, we must first understand the difference between short-term and long-term, and why price elasticities of demand for electricity might differ between the two time frames. We expect that energy use would differ in the short run in comparison to the long run. In the short run, customers are subject to the constraints placed upon them by the existing appliance stock, technologies, and infrastructure of their households. For example, if the price of electricity increases temporarily, as it might in warm summer months when air conditioning is in high demand, then a household might settle on a warmer air conditioner setting, or might opt to use cooling solutions that consume less electricity (fans, open windows, etc.). This result would show that the demand for electricity in the short run is elastic. In the long run, however, the appliance stock, technologies, and infrastructure of household are variable. Customers can react to long-term changes in price levels for electricity, buying purchasing more efficient appliances, for example. Therefore, long run elasticities incorporate both changes in the electricity utilization behavior of the residential consumers and any adjustments to the stock of appliances. However, the only problem with tracing price elasticities of demand for electricity of appliances in the long run is that we do not know the extent to which fluctuating electricity prices will prompt a consumer to replace appliances [15].

One major problem when we start looking into the price elasticity of demand for electricity, in general, is that households are remarkably different in the set of appliances they own, and, further, the modernity or quality of appliances that one household owns may vary significantly from the appliances of another household. For example, a household that has central air conditioning can exhibit a large price elasticity of demand for electricity as the household can simply increase the desired temperature of its central air conditioning if there is an increase in price, thus drastically reducing electricity consumption even with only a slight increase in price. On the other hand, a college household with a slim stock of appliances, perhaps only a fridge, oven, and a fan, might show little demand response to even a large change in prices, thus creating an inelastic price elasticity of demand for electricity.

However, we must also take into account that households that can afford to run a central air conditioner will be more wealthy than households that can only afford a fan; thus, the household that runs a central air conditioner may not react very much to changes in prices in comparison to a college student. As we can see from this discussion, comparing price elasticities of demand for electricity between households is quite difficult, and will yield values that are not necessarily accurate. Given the complexity and wide spectrum of types of households in the United States, it would be quite difficult to obtain a price elasticity of demand for electricity on a national scale that is completely accurate.

When we determined price elasticity of demand for electricity on a national scale, we simplified our electricity consumption function of the consumer to be: x* = x(p*). Thus, we do not consider the disparity of incomes, the different consumer behaviors, nor the willingness to wait for electricity. We assume the simple demand function because with regards to electricity on the whole, consumers are not going to be willing to not consume (total) electricity this month in favor of next month. We did not consider differences in income, at least here, because in the following model we analyze price elasticities of demand for electricity across different income brackets. We simply analyzed how electricity sales varied from month to month, and compared these sales to the fluctuations in price. We computed price elasticity of demand for electricity for year over year and for month over month [16].

Unfortunately, good time-series data on household electricity consumption is not available. The values we received when looking at the average price elasticity of demand for electricity for the United States, as a whole, were not consistent and varied widely. Although we believe that the range of values calls into question the significance of our calculations, we determined that the value for the average price elasticity of demand from 1995 to 2008 to be -0.87. What this means is that on average, when the price of electricity rises 1 percent, the demand for electricity will decrease by 0.87 percent. This result makes sense intuitively because as the price of electricity increases (on average), consumers will look to reduce their energy consumption, although their actions will be marginal (because of an inelastic value). However, as we just stated, this value must be taken lightly, given the relatively sparse data set on which the calculation was based.

We observe that, month over month since January 2007, the average price elasticity of demand is 0.27. This suggests that for every 1 percent increase in the price of electricity, demand for electricity also increases 0.27 percent. This result is a bit confusing, however, as month over month we would expect consumers to react readily to the price information they have available to them and would alter their consumption habits accordingly. However, on a month-to-month basis, we must understand that the warmer and colder seasons of the year will be stressed, as those months will show large values of *ε_d_*. On a yearly basis, electricity consumption is smoothed throughout the 12 months, thus no individual month is given special emphasis. Therefore, the range for *ε_d_* of year over year values is much smaller; the values are much more consistent. On a month over month basis, there is an extreme dispersion of *ε_d_* values. While we must understand that economically consumers want to react to higher electricity prices by reducing consumption of electricity, physically speaking they may be unwilling to change their consumption habits proportionately (if they still need heating or cooling in the cold or warm months, respectively).

Analyzing price elasticity of demand for electricity, we might expect that households of similar income would contain similar appliances of a similar type; thus, if we narrow our data for computing a price elasticity of demand to a single income bracket, the price elasticity of demand we calculate for each income bracket should be more unique.

As we see below, in Table 2, when we compute the price elasticity of demand for electricity over the different income brackets, we arrive at values that differ greatly, but intuitively make more sense, than when the estimated price elasticity of demand for electricity of the nation as a whole. However, the only data available on electricity consumption per income bracket was from two years, 1997 and 2001 [17]. Thus, while the following values may make sense according economic intuition, the conclusions are not necessarily well-supported, as a richer dataset would be necessary.

**Table 2:** Electricity demanded across income classes

2001	All households	< 10,000	10k – 29.9k	30k – 49.9k	50k or more
Total households (millions)	107	11	30.6	27.1	38.3
Billion kWh	1140	79	272	286	503
Average billion kWh per HH	10.65	7.18	8.88	10.55	13.13
Average annual price of electricity	8.58 cents per kWh				

**1997**	**All households**	**< 10,000**	**10k – 29.9k**	**30k – 49.9k**	**50k or more**

Total households (millions)	101.5	13.3	29.1	31.1	27.9
Billion kWh	1037	98	260	317	362
Average billion kWh per HH	10.22	7.37	8.93	10.19	12.97
Average annual price of electricity	8.4 cents per kWh				

**Computations**	**All households**	**< 10,000**	**10k – 29.9k**	**30k – 49.9k**	**50k or more**

% change in average billion kWh per HH	4.11%	-2.60%	-0.52%	3.42%	1.20%
% change in price	2.10%				
Price elasticity of demand	1.96	-1.24	-0.25	1.63	0.57

First of all, we see that our data is broken down into four income brackets, which we can classify as low income (<$10,000), low middle income, ($10,000-$29,999), high middle income ($30,000-$49,999), and high income ($50,000). It is necessary to understand the idea of disposable income and how it comes into play when analyzing electricity demands for the different income brackets. Disposable income is the amount of income left to an individual (or, in our case, a household) after taxes have been paid; thus, disposable income is money that an individual or a household has to use with at their discretion. So, the disposable income of a low-income household is much smaller than the disposable income of a high-income family; thus, a high-income family can afford luxuries that a lower income family cannot. For example, on luxury that a high-income family can afford is to use electricity even when the price of electricity goes up. Whereas a family with little disposable income will have to budget their electricity consumption better if the price of electricity prices goes up, high income families, with such large disposable incomes, will be more or less unaffected by a change in price of electricity as the increase in money spent on electricity will be a marginal percentage of their disposable income.

We see in Table 2 that the Price elasticity of demand for all households in the United States is 1.96, a figure that does not make much sense. This price elasticity of demand states that as price of electricity rises 1 percent then the demand for electricity also rises 1.96 percent, which, economically, is largely inexplicable. However, as we stated before, it is difficult to calculate a reasonable and accurate value for the price elasticity of demand on a national scale; this fact is due to the wide distribution of households, appliances in those households, and the behaviors of the consumers in those households.

Next, we see that the price elasticity of demand for electricity of households that earn $10,000 or less is -1.24, a value that shows that electricity demand is not only elastic, but the quantity change in demand of electricity is also inversely related to the quantity change in price. For these low income households, on average, if the price of electricity rises by 1 percent, than the average demand for electricity will decrease by 1.24 percent. Such a value makes sense because low-income families have very little disposable income; thus, a change in price of electricity will make the low income consumers alter their electricity consumption habits in order to make up for the increase in price.

For low middle-income families, the price elasticity of demand for electricity of households is -0.25, a result that shows that electricity demand is relatively inelastic. What this means is that for every 1 percent increase in the change in price of electricity, low middle-income families will consume, on average, 0.25 percent less electricity. This value also makes sense: Low middle-income families are obviously better off than low-income families, however, they still do not have much disposable income. Therefore, low middle-income families will still have to adjust their electricity consumption habits if the price of electricity increases, albeit to a lesser extent than low-income families. Recall that if the absolute value for the price elasticity of demand is less than one, the value is inelastic. Thus, we can say that for low middle-income families, their responsiveness to a change in price of electricity is relatively inelastic.

For high middle-income families, we compute a price elasticity of demand for electricity that is equal to 1.63, a value that does not make much sense economically. This value, although elastic, suggests that as the price of electricity goes up, demand for electricity in this income bracket also increases. The only way we can rationalize this statement would be that although the price of electricity increased from 1995 to 2001, consumers in this income bracket either accumulated more appliances or appliances which consume more electricity, without suspecting or caring about a possible increase in electricity prices.

Lastly, for high-income families, we calculated a price elasticity of demand of 0.53, a value that shows that electricity demand is relatively inelastic. While low middle-income families also expressed an inelastic price elasticity of demand for electricity, low middle-income families consumed less electricity as prices went up. A price elasticity of demand of 0.53 shows that high-income families will still consume more electricity as prices go up. However, we need to assume that over time a high-income family will accumulate more appliances and thus demand more electricity, and will not necessarily care about a change in price.

**Figure 2:**
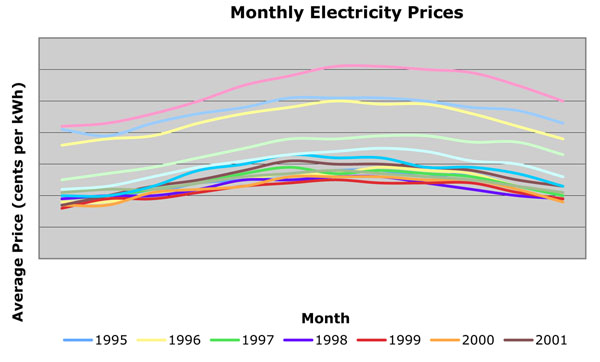
Electricity prices by month: 1995 – 2001

In a further attempt to control for seasonal effects, such as temperature and number of light hours per day, we can analyze the price elasticity of demand for electricity per month of year *t* over per month of year *t -1*. We figure that by looking at the same month, year over year, consumer behavior and demand for electricity should be similar, so their value for price elasticity of demand for electricity should be more telling of their true responsiveness to an increase or decrease in price for electricity. Hence, for example, we would analyze the changes in quantity demanded and price for January of 2008 over January 2007. Once we compute price elasticities of demand for electricity for each specific month from year 1995 to 2008, we averaged our values to produce the subsequent graph. As we see here, price elasticity of demand for electricity is more elastic, in the sense that consumers change their behavior the most to a change in price, in the hot summer months (especially July through August), and in the cold winter months. During these hot summer months, especially, air conditioning is used extensively to control the temperature inside the household. Due to the high demand for air conditioning, during the hot summer months, we see that electricity prices are highest during these times of year, as shown in Figure 2. The historically high prices of electricity prices justify the largest value for price elasticity of demand for electricity to occur during these months, as that is when a change in price will affect the consumer the most.

However, air conditioning and heating use a lot of electricity; thus, if the price of electricity goes up more than expected, they can become costly appliances. We especially see that in the hot summer months of July and August, an increase in price of electricity will lead to consumers changing (reducing) their electricity consumption (air conditioning, in particular), thus creating the more elastic price demand.

Research on household responsiveness to changes in energy prices remains far from complete. Obstacles are encountered frequently due the power that authorities hold over any changes in the level of electricity pricing. Thus, determining the effect of a mild price signal in relation to the demand for other sources in play is often challenging and questions the validity and reliability of the data. However, the electricity industry restructuring plan that permitted authorities, in 1998 in California, to loosen their tight control over wholesale electricity prices and capacity decisions due to the abnormally lengthy periods of tight supplies in the wake of the California crisis led to a unique study assessing consumer reactions on the household level to tight supply conditions both when price freely vary and when prices were capped by Reiss and White [18]. As prices rose in the summer of 2000, from mid-July on average residential electricity consumption began to decline to the extent that electricity consumption decreased an overall 12 to 13 percent compared to previous years, controlling for weather. Evidence from data shows consumers failed to anticipate these higher prices and the decline in consumption was realized a full month after prices began their rapid rise – estimating a short time interval of approximately 60 days for the 12 to 13 percent drop in consumption to occur. In addition, given, that the imposition of the price cap in early September was coincidental with the reverse course of consumption behavior, strongly suggests that consumers responded to the sharp rise and the proceeding fall in electricity prices during 2000.

The analysis of household electricity consumption data in San Diego during a period when households experienced retail price changes reveal that consumers have extensive control over the short run electricity rate of their appliance use. Furthermore, these findings are applicable to current energy policy discussion and to the reform of electricity pricing. Contrary to the observation put forward by both Borenstein [19] and Wolak [20] that electricity demand from the residential point of view is insensitive to the actual cost of producing power at any point in time but need not necessarily embody consumer preferences, the San Diego data on one hand fails to verify whether responses on the demand side were statistically significant. On the other hand, it successfully refutes any views that consumers fail to respond to changes in short run electricity prices. Therefore, this suggests that more flexible pricing due to the implementation of a “real-time” pricing system would lead to higher degree of responsiveness in demand and, in turn, improved market efficiency. Although the benefits of such a policy are visible, the level of its efficiency will rest on the magnitude of the willingness of the consumers to curtail their electricity consumption over short-term horizons in response to high prices.

Incorporating the price elasticity generated for the demand of electricity according to the different income brackets, we can suppose that real time pricing will be most effective with households of low income, and second most effective with households of low middle income. We can make this determination because these are households (according to the data) that change their electricity consumption behavior the most in response to changes in the price of electricity.

We were unable to calculate the price elasticity of demand at the level of appliance energy consumption due to lack of the necessary data. We anticipate that analysis at this detailed level would be extremely valuable both in predicting real-time electricity consumption, and generally understanding the dynamics of real-time energy consumption and its effect on production. While we have yet to collect the necessary data, we develop a real-time pricing model below that can be tested when appliance-level data is available.

### Real time pricing model

Because it is difficult to quantify the degree to which consumers will respond to smart meter installation, we have developed a theoretical model to explore how household demand for electricity will respond to smart meter installation. As mentioned, a key attribute of a smart meter is its ability to accurately monitor the time of electricity consumption in addition to the quantity. This vast increase in real-time information delivered to the utility has a number of key consequences. First, the utility has the ability to introduce a real-time pricing scheme, based both on the demand for and availability of electricity at that time. Real-time pricing consists of a variable pricing system, which the utility is able to adjust in response to changes in demand on the system. Second, the consumer is able to react easily to changes in the price of electricity since the variable price will be displayed in the household. In this way, we expect to see an increased price elasticity of demand among households which both have smart meters and are subject to real-time pricing.

As there is insufficient data on consumer consumption of electricity under a real-time pricing scheme, we have examined general consumer consumption behavior in response to price changes. While there is generally little correlation between monthly price and monthly electricity consumption in the United States, some interesting conclusions can be drawn from the San Diego energy crisis in 2000-2001. Because this crisis involved rapid and substantial changes in price, this period offered an opportunity to analyze the relationship between the price of electricity and household consumption. A study conducted in 2003 by Peter Reiss of Stanford and Matthew White of Wharton indicates that there was a significant consumer response to changes in the price of electricity.

This study indicates that households tend to alter electricity consumption over a period of time in response to the cost of the previous electricity consumption. For example, the study found that after a substantial price increase in the summer of 2000, household electricity consumption decline substantially over the 60 days, approximately 12 to 13% on average. They write, “Overall, the results indicate consumers may be far more responsive to pecuniary and non-pecuniary incentives for altering their energy use than is commonly believed” [21]. With this in mind, we believe that smart meter installation will cause the consumer to become more responsive even to small changes in price, since they have real-time pricing data immediately available. By providing the household with consumption and pricing information, the smart meter will enable the household to more actively manage its electricity consumption.

This model is based on the assumption that the demand for electricity varies across different types of appliances. That is, we assume consumers have a different price elasticity of demand for use of different types of appliances. There is a fundamental difference in the way each type of appliance consumes electricity. Suppose there is a sudden increase in the price of electricity during the day. We expect that the quantity of electricity demanded for a refrigerator would be relatively constant, whereas the quantity of electricity demanded for a dishwasher, which can be programed to run in off-peak hours, would likely reduce significantly. Because of this difference, we divided common household appliances into 3 categories: passive, active & time-delay. In order to reflect both increasing technology and the ability of the consumer to use certain appliances during off-peak hours, we have included appliances in the “time-delay” category that do not necessarily have a time-delay function in every household.

Table 3 lists the common household appliances according to category and includes the current relative weight of each category on the total household consumption of electricity. This data is provided by the Energy Information Administration for U.S. electricity consumption in 2007.

**Table 3:** Electricity use across typical household appliances

Appliance/category	Electricity use
**Acitive appliances**	**28.42%**
Cooking	2.32%
Lighting	15.37%
Color television & related	7.58%
Personal computers	3.16%

**Passive appliances**	**54.32%**
Space heating	5.89%
Space cooling	18.53%
Refrigeration	8.21%
Freezers	1.68%
Furnace fans and boiler circulation	2.74%
Other uses	17.26%

**Time delay appliances**	**17.26%**
Water heating	8.84%
Clothes dryers	5.68%
Clothes washers	0.63%
Dishwashers	2.11%

**Total**	**100.00%**

We make several key assumptions about the factors affecting each category of the household demand for electricity, including correlation with price and correlation with the number of people at home and awake. The following consumption categories are listed in order of increasing price elasticity: passive; active; time-delay. That is, given an increase in real-time price, we expect the consumer will reduce his time-delay electricity consumption more than he will reduce his active electricity consumption. For example, a consumer would be more willing to delay running his dishwasher at 2:00AM than to wait to use his television at 2:00AM. The following consumption categories are listed in order of increasing correlation to *N*(*t*): active; passive; time-delay. This is relatively evident, since active appliances are in use only when people choose to use them. Passive appliances, such as a furnace, are semi-correlated to *N*(*t*), whereas time-delay appliances are correlated only to a very small degree.

We propose a theoretical model, the parameters of which have yet to be estimated, as there is insufficient data on the real-time electricity consumption patterns of different appliance categories. However, we feel that we have a reasonable sense of the consumer’s behavior such that we can estimate how the parameters differ across the three appliance categories. The household demand for electricity for each appliance category is as follows *J*(*t*) is the category, *y* is the household income, *P*(*t*) is the real-time price function and *N*(*t*) is the function corresponding to the number of people at home and awake. as The *ε_J_* term corresponds to the portion of demand that is related to other factors, such as weather conditions.

**Figure 3:**
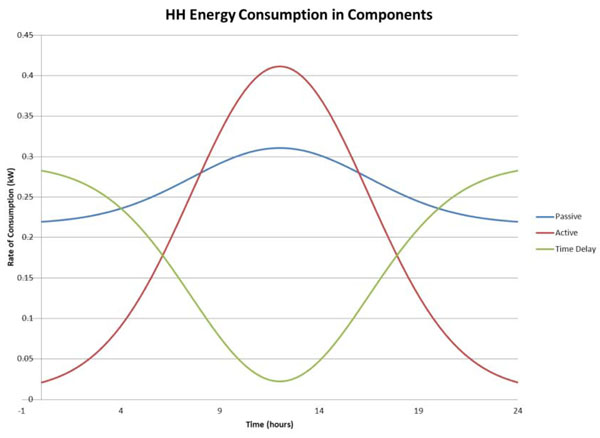
Hourly household energy consumption by appliance category

**Figure 4:**
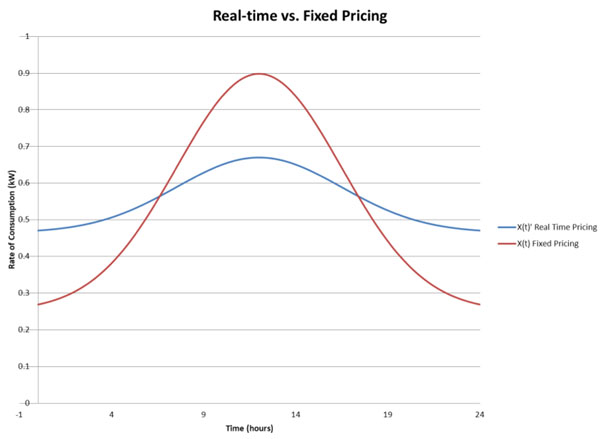
Effect of real-time pricing on hourly energy consumption

*J*(*t*) is the demand for an appliance category, *y* is the household income, *P*(*t*) is the real-time price function and *N*(*t*) is the function corresponding the number of people at home and awake. *J*(*t*) corresponds to the passive electricity demand, *K*(*t*) corresponds to the active electricity demand and *L*(*t*) corresponds to the time-delay electricity demand. Thus, we write total demand as follows:

The Utility then implements a real-time pricing scheme, which is correlated to *N*(*t*). In reality, the utility will be able to make price changes in response to changes in the demand for electricity. For simplicity, we assume that *N*(*t*) is derived from a normal distribution. This model projects household electricity consumption in real-time, under two scenarios. In the base case, we estimate consumption over 24 hours, with a constant pricing scheme. In the second case, we estimate consumption with a real-time pricing scheme. When real-time pricing is implemented, we predict that household consumption in each appliance category will resemble the pattern exhibited in Graph 1. Notice that consumption in the time-delay category shifts away from peak-hours to off-peak hours under real-time pricing. The sum of these three functions results in the real-time demand *X*(*t*)*_r_* with real-time pricing, whereas  represents real-time demand under constant pricing. These functions are depicted in Graph 2, where it is clear that real-time pricing reduces peak electricity consumption, and we see that consumption is shifted from the peak periods to off-peak periods. Figure 3 shows the correlation of household income with utility use. Figure 4, a complement to Fig. 3, shows the relation between energy pricing and hourly consumption of energy.

This model is intended to provide a better sense of how the household manages electricity consumption. As the number of appliances with time-delay features increases, the household has a greater ability to react to real-time pricing. In addition to a shift in consumption, most research on pilot smart-meter projects suggests that consumers will reduce their total electricity consumption as well. Thus, not only will consumption be smoothed across the day, but the amount of electricity consumed will be reduced. An article written in the published in the *Financial Times* considers a number of studies which concluded that the reduction in electricity consumption could range from 1%-15%. Most of the estimates were close to 5%, so this is the number assumed in the following benefit analysis.

Another benefit comes from the fact that less electricity will need to be produced. Presently, many generation facilities are running perpetually at full capacity to cover peak demand. With a smoothed consumer demand function, we expect that utilities will be able to run at a lower capacity and a larger proportion of the produced electricity will actually be consumed by households. In the benefit analysis, we assume that larger ratio of (*consumed electricity*)/(*produced electricity*) will lead to a reduction in the average market price for electricity, once smart meters are installed. This is due to an expected decrease in the average costs of production, since less electricity will be produced at facilities.

### Cost-benefit analysis

In order to make a more precise estimate of benefits, we chose to narrow the analysis to the installation of smart meters in residential households in the United States. This analysis omits key infrastructure upgrades to the current electric grid in the United States, including the replacement and addition of transmission lines and distribution stations. This analysis also omits the implementation of “smart sensors” which can be installed at key locations on the grid to monitor transmission as well as system problems. These sensors would add to the reliability of the grid by reducing blackout frequency and duration. However, our goal in this analysis is to produce a conservative estimate for the direct benefits of only implementing smart meters in every U.S. household. We conclude that smart meters alone will significantly improve the electric grid, without the need for immediate infrastructure changes. This is not to say the infrastructure does not need to be improved, rather that efforts should be initially focused on achieving the short-term benefits of smart meter implementation.

The benefit analysis largely depends on our assumptions about electricity consumption over the next 15 years as well as the average price of electricity. First, we assume that that there will be a sharp decrease in electricity consumption within the first year, following implementation. Not only do we anticipate that households will shift consumption from peak hours to off peak hours, but also that a more conscious consumer will reduce his overall electricity consumption. Because the household will see its rate of consumption at any given time, it will become aware of which appliances use more electricity and how it can efficiently reduce consumption. We assume that overall consumption will decrease by 5 percent in the first year following smart meter installation. This assumption is fairly conservative and consistent with research estimating the potential reduction in energy consumption due to smart meter implementation. This research includes recent studies by Oxford’s Environmental Change Institute and General Electric [22]. In the following years, we estimate that consumption will continue to grow at 0.8 percent, which is consistent with the EIA’s projection for electricity consumption through 2030. We think this is a very conservative estimate, since we expect that smart meter implementation will reduce this rate of growth as average households attempt minimize consumption.

In addition to a reduction in overall consumption, we also assume that the average price for electricity will decrease in the first year following implementation. Because smart meters will reduce the peak load on the system and spread consumption to other periods of the day, utilities will be able to cover peak demand at a lower level of production. They will also be able to adjust output, according to the real-time consumption data provided by the smart meters. As we mentioned earlier, we estimate that the average cost of electricity production is $0.06 per kWh in the United States. If utilities can reduce the average cost by 10 percent, we assume that the average price will reduce by approximately 5 percent. This estimate is based on the average price from 2009 through August, which is $.1136 per kWh. We assume that prices will continue increase by 0.006 percent, which is also consistent to EIA projections through 2030. In order to calculate the benefits in each year following implementation, we multiply projected total U.S. consumption in each year by the projected average price in that year. Based on the reduced electricity consumption, we also estimate that CO_2_ costs to the environment will be reduced. We calculated this by assuming a cost of $25 per metric ton of CO_2_ and used the EIA estimate for CO_2_ produced per unit of generated electricity in the United States.

The remaining key assumptions include reductions in congestion rent and blackout costs in the United States. As defined in the cost analysis portion of the paper, congestion rent is total cost associated with attempting to an amount of electricity through the transmission system that is greater than the capacity of the transmission lines. As mentioned earlier, this cost was estimated to be $8.36 billion for 2009. We predict that utilities will be able to adjust prices during to reduce consumption during these periods of excess demand on the system. Due to this ability and smoothed household demand, we estimate that costs associated with congestion rent will be largely eliminated by smart meter implementation. Thus, we assume that 90 percent of congestion rent will be eliminated. Blackout costs for 2009 are estimated to be $150 billion. Because the frequency of blackouts depends on a number of factors, which can also be reduced by upgrading the infrastructure, we predict that 15% of blackout costs will be reduced with nationwide smart meter implementation. While improvement to the transmission infrastructure will likely add to the reduction in blackout costs, we expect that smart meters will significantly reduce the overall blackout-causing strain on the system.

The costs associated with smart meter implementation are relatively simple, and are based on detailed analysis conducted by in the United Kingdom by *Sustainability First*. We see that their findings are consistent with much of the other analysis on the costs of smart meters, but their estimates include more advanced smart meter features including a digital display, which is necessary for effective real-time pricing. Their analysis also includes costs for installation as well as infrastructure changes at utilities in order to monitor consumption and the real-time pricing system. We assume that reduced maintenance costs for utilities will balance with the costs of managing the real-time pricing system. *Sustainability First* predicts that the total initial capital costs for installation of an advanced smart meter would range between £123-180 including separate display [23]. As a conservative estimate, we assumed a smart meter capital cost $250 per meter, which will be paid upfront in the year of installation. We also assumed a maintenance cost of $12.5 per meter per annum, which is consistent with *Sustainability First*’s predictions.

**Figure 5:**
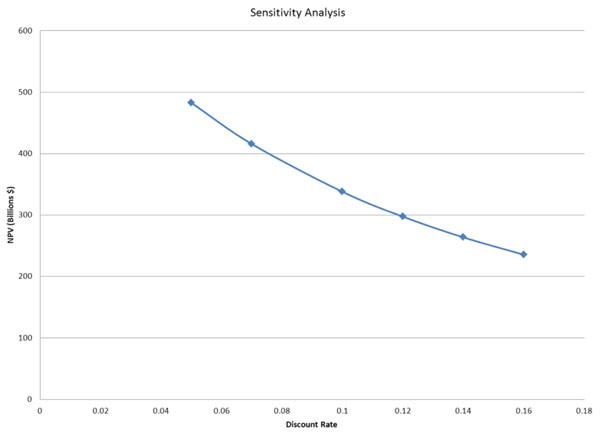
Cost-Benefit calculation sensitivity analysis

Finally, in order to estimate the net benefit of nationwide smart meter installation in the United States, we calculated the present value of the potential net benefits. The present value is based on a 15 year time horizon, which is a conservative estimate for the life of a smart meter. Because we included savings to the economy from reduced blackouts as well as environmental benefits, we assume a 7% discount rate. This rate is largely based on our expectation that smart meter implementation will require investment and support on the part of utilities and the U.S. government. We also show the sensitivity of our result due to changes in the discount rate in Figure 5, in order to provide a sense of how the consumer could consider the benefits of smart meter implementation. We calculate an estimated benefit in net present value of approximately $436 billion, after costs of approximately $45 billion. This conclusion highlights the relatively low cost of smart meter implementation, as well as the substantial potential benefits to households, the economy and the environment.

## Conclusion

We conclude that the United States should formulate a comprehensive and near-term plan to install smart meters nationwide. Because smart meters rely largely on communication between the utility and the household through the current electricity transmission system, it is not necessary to simultaneously implement large-scale infrastructure changes. While it is important to plan comprehensive infrastructure improvement over the coming decade, these infrastructure projects will complement the benefits that smart meters offer both in the near future and for years to come.

## Competing interests

The authors declare that they have no competing interests.
